# Interactions between vitamin D deficiency and inflammation on diabetes risk: data from 336,500 UK Biobank adults

**DOI:** 10.1016/j.jnha.2024.100446

**Published:** 2024-12-10

**Authors:** Jatupol Kositsawat, Shangshu Zhao, George A. Kuchel, Lisa C. Barry, Richard H. Fortinsky, Ben Kirk, Gustavo Duque, Chia-Ling Kuo

**Affiliations:** a Center on Aging, University of Connecticut, Farmington, CT; b The Cato T. Laurencin Institute for Translation in Regenerative Engineering, University of Connecticut Health, Farmington, CT, United States; c Department of Medicine, Western Health, Melbourne Medical School, University of Melbourne, St Albans, Melbourne, VIC, Australia; d Australian Institute for Musculoskeletal Science (AIMSS), University of Melbourne and Western Health, St Albans, Melbourne, VIC, Australia; e Bone, Muscle and Geroscience Group, Research Institute of the McGill University Health Centre, Montreal, QC, Canada; f Dr. Joseph Kaufmann Chair in Geriatric Medicine, Department of Medicine, McGill University, Montreal, QC, Canada

**Keywords:** Vitamin D deficiency, Inflammation, Frailty, Aging, Diabetes risk

## Abstract

**Objectives:**

Findings regarding the effects of vitamin D supplementation on diabetes risk are inconclusive. Because inflammation and vitamin D levels are interconnected, we hypothesized that higher inflammation levels moderate the effects of vitamin D deficiency on diabetes risk.

**Design, setting, participants, and measurements:**

UK Biobank participants without pre-existing diabetes at baseline were included (N = 336,500). We first linked vitamin D and C-reactive protein (CRP; inflammation measure) levels with incident diabetes during a mean follow-up of 13.5 years (SD = 1.9). Then, we investigated the moderation effect of CRP on the associations between vitamin D deficiency (<10 ng/mL) and incident diabetes and performed subgroup analyses according to age (<60 vs. ≥60 years) and frailty status (frail; pre-frail; non-frail). Multivariate analyses were conducted using restricted cubic spline Cox proportional hazards regression models.

**Results:**

Lower vitamin D and higher CRP levels were significantly associated with an increased risk of diabetes during follow-up. There was a significant interaction between vitamin D deficiency and CRP on incident diabetes (p < 0.001). In participants with higher levels of CRP, the hazard ratio of developing diabetes comparing participants who had vitamin D deficiency to those who did not was lower than that in participants with lower levels of CRP. The moderation effect of CRP was similar between younger and older adults but was stronger in frail or pre-frail older adults than in non-frail older adults.

**Conclusion:**

Our findings indicate that the effect of vitamin D deficiency on incident diabetes may be affected by inflammation. This finding may explain the inconsistent results from vitamin D supplementation trials. Vitamin D supplementation without considering the potential impact of inflammation might prove unsatisfactory.

## 1 Introduction

Diabetes mellitus has become increasingly prevalent. It was declared an epidemic in 1994 by The Centers for Disease Control and Prevention (CDC). In 2021, 38.4 million Americans (11.6% of the U.S. population) and 16.5 million seniors (29.2 % of adults ≥65 y) had diabetes. Additionally, 97.6 million Americans (38% of adults ≥18 y) have prediabetes and 1.2 million Americans are newly diagnosed each year [1]. This condition significantly impacts health and causes a tremendous socioeconomic burden in the U.S. with an estimated cost of 412.9 billion dollars in 2022 [2]. Thus, primary prevention is of utmost importance to slow down the diabetes epidemic.

Previous observational studies report that vitamin D deficiency is associated with poor glycemic control [3,4] and increased risk of mortality [5] in patients with diabetes. However, findings from several clinical trials evaluating the effect of vitamin D replacement on diabetes and described in systematic reviews are equivocal; some have reported a beneficial effect of vitamin D replacement on preventing the transition from prediabetes to diabetes [6,7], while others have not found any benefit [8]. Various explanations for the mixed results from supplementation trials may include a single unified dosage of vitamin D supplementation and baseline characteristics of participants that may affect the response to supplements, including older age, race/ethnicity, obesity, and genetic factors, among others [[9], [10], [11], [12]].

Another possible explanation for these mixed findings is the role of inflammation. Previous reports showed an association between inflammation and diabetes [13,14] and proposed that targeting inflammation may help to prevent and control diabetes. Relatedly, vitamin D deficiency has been shown to be associated with increasing inflammation both in vitro and in vivo [15,16], and correcting such deficiency may improve inflammatory states [17].

As described above, inflammation, vitamin D deficiency, and diabetes are intricately connected. Evidence that supports such connection also includes seasonal variation of vitamin D levels and glycemic control, involvement of vitamin D status in pathways of developing diabetes, and influence of vitamin D on glucose homeostasis through modulating inflammatory pathways [18]. Consequently, managing one condition without considering other inter-connected factors will likely not lead to achieving desirable outcomes. In addition, personalized medicine, which emphasizes individualizing care for each person and tailoring treatments to meet specific needs, is needed to maximize benefits and minimize side effects.

Notably, frailty, a state of diminished reserve that leads to increased susceptibility to adverse clinical outcomes in response to physiological stress [19,20], is also closely related to diabetes. A systematic review and meta-analysis proposed that frailty identification and assessment should be a part of routine diabetes care [21]. In older adults, frailty was shown to be associated with an increasing risk of developing incident type 2 diabetes [22]. Frailty was also associated with multimorbidity and mortality [23].

In this study, to shed light on inconsistent results from vitamin D supplementation trials, we hypothesized that inflammation reduces the effects of vitamin D deficiency on incident diabetes in middle-aged and older adults. We also explored whether such interactions of vitamin D deficiency and inflammation on diabetes risk are found in subgroups defined by age and frailty status, two important risk factors associated with diabetes outcomes and complications.

## 2 Methods

Between 2006 and 2010, more than 500,000 UK Biobank participants aged 40–70 years underwent physical measurements and completed online questionnaires about their medical history, lifestyle, and sociodemographics [24]. Disease diagnoses and mortality during a mean follow-up of 13.5 years (SD = 1.9) were updated through linkages with electronic health records [24].

Participants without a diabetes diagnosis in their electronic health records at baseline were considered to be diabetes-free. After excluding those with any missing values of vitamin D, CRP, or baseline covariates (described below), a total of 336,500 participants without pre-existing diabetes at the study baseline were included in this study (Supplementary Fig. S1). At baseline, serum vitamin D levels were assessed using Clinical Laboratory Improvement Amendments (CLIA) analysis performed on a DiaSorin Ltd. LIASON XL instrument. Serum C-reactive protein (CRP) levels were measured using immunoturbidimetric - high sensitivity analysis conducted on a Beckman Coulter AU5800 analyzer. Please consult the UK Biobank Biomarker Assay Quality Procedures for specific technical information regarding blood chemistry analyses [25].

Participants diagnosed with diabetes (ICD-10 codes E10, E11, E12, E13, E14) during follow-up were identified using the UK Biobank first occurrence data, which integrates multi-source data based on ICD-10 codes (primary care, hospital inpatient, death register records, and self-reported medical conditions at baseline). We used the censoring date as the date of death (up to November 30, 2022) or the last follow-up date of hospital inpatient records, depending on which occurred first. The last follow-up date of hospital inpatient records varied with countries: October 31, 2022, for participants visiting an assessment center in England, August 31, 2022, for participants in Scotland, and May 31, 2022, for participants in Wales.

The UK Biobank first occurrence data were also used to identify any major adverse cardiovascular events (MACE; ICD-10 codes I20-I25, I61-I64, I70, I73) and chronic kidney disease diagnosis (N18) before or at baseline. These variables were included as covariates. Other baseline covariates included self-reported demographic, socioeconomic and lifestyle factors, (1) demographic factors: chronological age, sex (male or female), ethnicity (White, Black, Asian, or Other), season at recruitment (Spring: March to May, Summer: June to August, Fall: September to November, Winter: December to February); (2) socioeconomic factors: Townsend deprivation index (higher values indicating higher levels of material deprivation), education qualification (from college or university degree to none of the listed degrees); (3) lifestyle factors: smoking status (never, previous, or current smoker), alcohol intake frequency (from daily or almost daily to never), physical activity group, measured by the short International Physical Activity Questionnaire (IPAQ) [26], and vitamin D supplement (yes/no, assessed by online questionnaire). Additionally, we included body mass index (BMI). BMI was calculated from weight in kilograms divided by the square of height in meters. The Fried frailty criteria were used to determine frailty status (non-frail, prefrail, frail) (19) for subgroup analysis. Participants were considered non-frail if none of the following items were endorsed, prefrail if 1–2 items were endorsed, and frail if three or more items were endorsed: (1) self-reported weight loss (yes/no, based on a survey question to ask weight change compared to one year ago), (2) exhaustion (yes/no, based on a survey question to ask frequency of feeling tired or having little energy over the past two weeks), (3) self-reported slow walking pace (yes/no, based on a survey question to ask usual walking pace: slow if less than 3 miles per hour), (4) lowest 20% of maximal hand grip strength in the same sex group (yes/no), (5) lowest 20% of physical activity in the same sex group (yes/no), by the short version of IPAQ. A list of UK Biobank field IDs used to extract data is provided in Supplementary Table S1.

A descriptive analysis was conducted to summarize variables using proper statistics overall and for participants who developed diabetes during follow-up versus those who did not. For preliminary analyses, each variable was compared between the two groups of participants using a linear Cox regression model. Next, vitamin D and CRP were transformed into z-scores using the inverse normal transformation to correct the distributional skewness and unify the scales. The relationship between vitamin D z-scores and incident diabetes was modeled using a Cox regression model for time from baseline assessment to first diagnosis of diabetes, including a restricted cubic spline function of vitamin D (knots at 0.1, 0.5, and 0.9 quantiles) and covariates. As a result, the adjusted hazard ratio (HR) comparing a vitamin D z-score to the mean vitamin D z-score was plotted. The HRs associated with the vitamin D z-scores of −2, −1, 0, 1, and 2 were tabulated, including the corresponding vitamin D levels in the original scale. These methods were also applied to model the association between CRP and incident diabetes.

We then investigated the moderation effect of CRP on the association between vitamin D deficiency (vitamin D levels below 10 ng/mL) and incident diabetes using a non-linear Cox regression model. In addition to vitamin D deficiency, the model included a restrictive cubic spline function of CRP z-score (knots at 0.1, 0.5, and 0.9 quantiles), the product of vitamin D deficiency and the CRP z-score function, and covariates (interaction model). Based on the fitted model, the adjusted HR associated with vitamin D deficiency given a CRP z-score was plotted, including the numerical results at the CRP z-scores of −2, −1, 0, 1, and 2. Overall, the interaction between vitamin D deficiency and CRP was tested by comparing the interaction model to its reduced model without the vitamin D deficiency and CRP interaction term using an ANOVA F-test.

Subgroup analysis was conducted by age group (younger adults <60 y or older adults ≥60 y) and by frailty status in older adults (non-frail or pre-frail/frail) at baseline. All hypothesis tests were two-sided and statistical analyses were performed in R version 4.3.2 using R packages including “survival” and “rms”.

## 3 Results

Most of the sample (n = 336,500) identified their race as White (96%, Supplementary Table S2). At baseline, 13 % had vitamin D deficiency (25-OH vitamin D levels <10 ng/mL, Supplementary Table S2) and a total of 16,423 participants (4.9 %) developed diabetes during follow-up (Table 1). Male sex, older age, and non-White ethnicity were associated with a higher risk of incident diabetes. Participants with lower socioeconomic status, unhealthy lifestyles, or those who were frail or previously diagnosed with CKD or MACE had a higher risk of developing diabetes during follow-up. Of note, vitamin D supplement use was not significantly associated with incident diabetes.

**Table 1:** Baseline participant characteristics of the included sample by the status incident diabetes during follow-up.

	Incident diabetes		
Variable	No, N = 320,077^1^	Yes, N = 16,423^1^	P-Value^2^
Exposure			
C-reactive Protein (mg/L)	2.36 (4.11); 1.21 (0.60, 2.47)	4.01 (5.27); 2.34 (1.19, 4.67)	<0.001
Vitamin D (ng/mL)	20 (8); 19 (13, 25)	17 (8); 16 (11, 22)	<0.001
Vitamin D Deficiency (<10 ng/mL)			<0.001
No	281,238 (88%)	13,138 (80%)	
Yes	38,839 (12%)	3,285 (20%)	
Demographic Factors			
Age at recruitment	56 (8); 57 (49, 63)	59 (8); 60 (53, 65)	<0.001
Age Group			<0.001
<60	191,371 (60%)	7,563 (46%)	
≥60	128,706 (40%)	8,860 (54%)	
Season at Recruitment			<0.001
Spring	91,972 (29%)	4,652 (28 %)	
Summer	84,590 (24%)	4,526 (28%)	
Fall	78,310 (26%)	3,837 (23%)	
Winter	65,205 (20%)	3,408 (21%)	
Sex			<0.001
Male	149,641 (47%)	9,823 (60%)	
Female	170,436 (53%)	6,600 (40%)	
Ethnicity			<0.001
White	306,736 (95.8%)	14,919 (90.8%)	
Asian	5,119 (1.6%)	687 (4.2%)	
Black or Black British	3,989 (1.2%)	507 (3.1%)	
Other	4,233 (1.3%)	310 (1.9%)	
Socioeconomic Factors			
Townsend Deprivation Index	−1.50 (2.97); -2.30 (-3.72, 0.18)	−0.69 (3.36); -1.53 (-3.35, 1.56)	<0.001
Education^3^			<0.001
None of the above	43,360 (13.5%)	4,089 (24.9%)	
Other professional qualifications e.g.: nursing, teaching	15,967 (5.0%)	977 (5.9%)	
A-levels/NVQ/HND/HNC	58,778 (18.4%)	3,061 (18.6%)	
GCSEs/O-levels	68,460 (21.4%)	3,503 (21.3)	
CSEs or equivalent	16,765 (5.2%)	852 (5.2%)	
College or University degree	116,747 (36.5%)	3,941 (24.0%)	
Lifestyle Factors			
Vitamin D Supplement Status			0.37
No	314,241 (98.2%)	16,143 (98.3%)	
Yes	5,836 (1.8%)	280 (1.7%)	
Smoking Status			<0.001
Never	178,884 (56%)	7,470 (45%)	
Previous	109,738 (34%)	6,590 (40%)	
Current	31,455 (10%)	2,363 (15%)	
Alcohol Frequency			<0.001
Never	21,119 (6.6%)	1,944 (12%)	
Special occasions only	31,854 (10%)	2,643 (16%)	
One to three times a month	34,624 (11%)	2,018 (12%)	
Once or twice a week	83,543 (26%)	4,024 (25%)	
Three or four times a week	79,274 (25%)	3,039 (19%)	
Daily or almost daily	69,663 (22%)	2,755 (17%)	
International Physical Activity Questionnaire (IPAQ) Activity Group			<0.001
Low	57,127 (18%)	4,163 (25%)	
Moderate	130,863 (41%)	6,537 (40%)	
High	132,087 (41%)	5,723 (35%)	
Physical Measures			
BMI	26.9 (4.4); 26.3 (23.9, 29.2)	31.0 (5.4); 30.3 (27.3, 34.0)	<0.001
Frailty (age ≥ 60 only)			<0.001
Frail or Pre-Frail	51,197 (42%)	4,521 (56%)	
Non-frail	69,725 (58%)	3,520 (44%)	
Disease Status			
Major Adverse Cardiovascular Events Status			<0.001
No	300,689 (94%)	13,742 (84%)	
Yes	19,388 (6%)	2,681 (16%)	
Chronic Kidney Disease Status			<0.001
No	317,200 (99%)	16,075 (98%)	
Yes	2,877 (1%)	348 (2%)	

1 Mean (SD); Median (IQR) or Frequency (%).

2 Based on Cox regression models.

3 Education: CSE - Certificate of Secondary Education; GCSE - General Certificate of Secondary Education; O-levels - Ordinary Levels; A-levels - Advanced Levels; NVQ - National Vocational Qualification; HND - Higher National Diploma; HNC - Higher National Certificate.

**Fig. 1:**
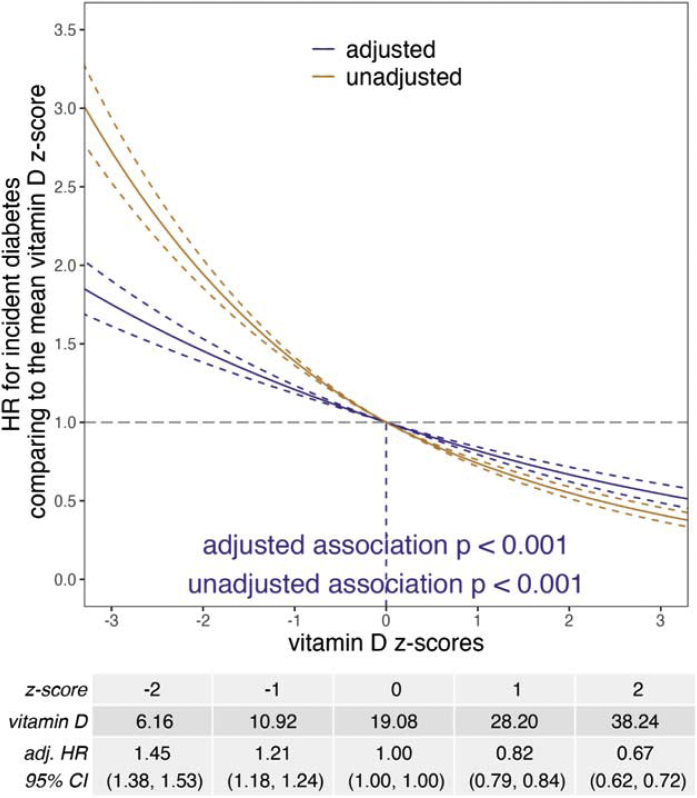
Unadjusted and adjusted hazard ratios (HRs) for incident diabetes comparing a vitamin D z-score to the mean vitamin D z-score (approximately 0). Adjusted baseline covariates: age, sex, ethnicity, recruitment season, education, Townsend deprivation index, smoking status, IPAQ activity group, alcohol intake frequency, BMI, vitamin D supplement status, MACE status, and chronic kidney disease status.

**Fig. 2:**
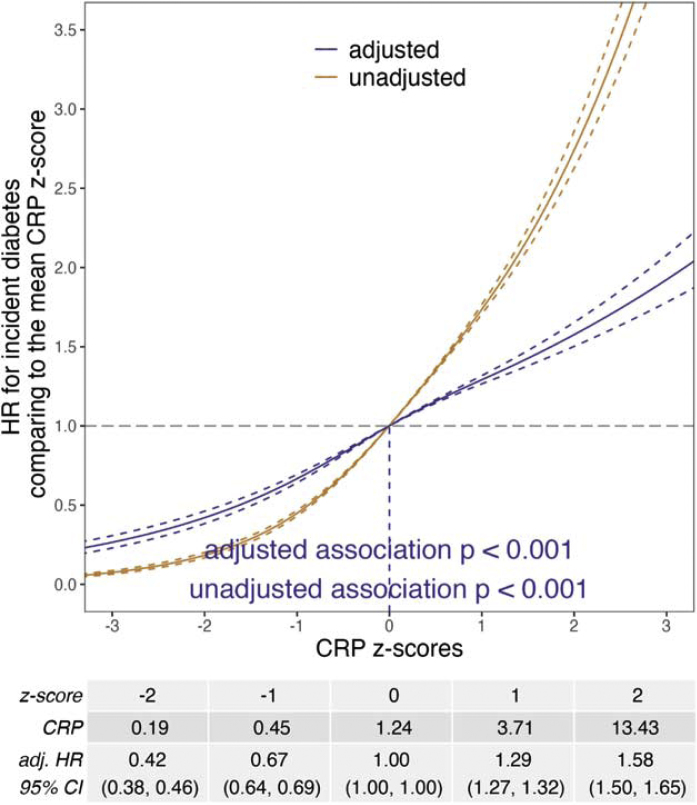
Unadjusted and adjusted hazard ratios (HRs) for incident diabetes comparing a CRP z-score to the mean CRP z-score (approximately 0). Adjusted baseline covariates: age, sex, ethnicity, recruitment season, education, Townsend deprivation index, smoking status, IPAQ activity group, alcohol intake frequency, BMI, vitamin D supplement status, MACE status, and chronic kidney disease status.

**Fig. 3:**
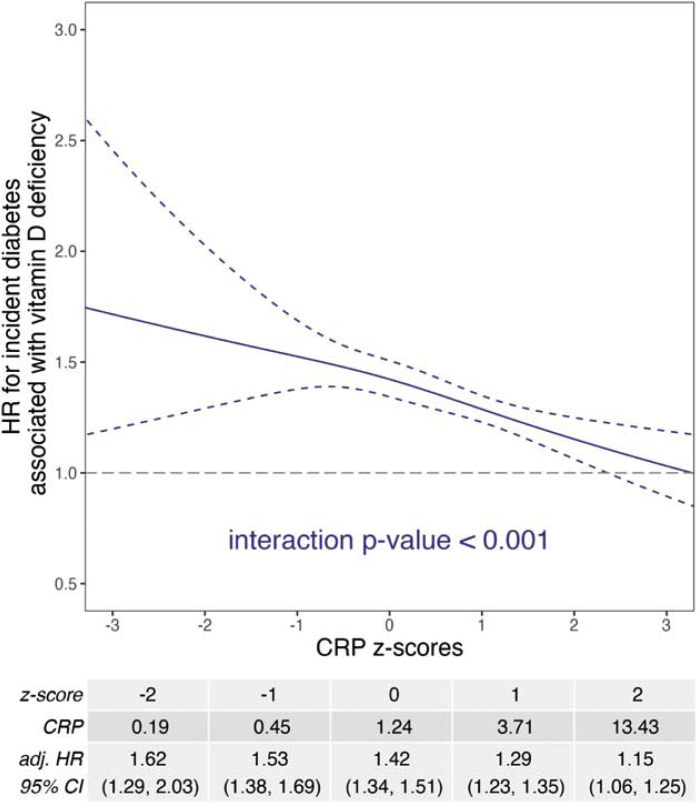
Adjusted hazard ratios (HRs) for incident diabetes associated with vitamin D deficiency at a given level of CRP z-score. Adjusted baseline covariates: age, sex, ethnicity, recruitment season, education, Townsend deprivation index, smoking status, IPAQ activity group, alcohol intake frequency, BMI, vitamin D supplement status, MACE status, and chronic kidney disease status.

Participants who developed diabetes during follow-up had a lower mean vitamin D level (17 ± 8 vs. 20 ± 8 ng/mL) and a higher mean CRP level (4.01 ± 5.27 vs. 2.36 ± 4.11 mg/L) than those who did not (Table 1). The associations between vitamin D or CRP and incident diabetes remained statistically significant after adjusting for covariates (p < 0.001, Fig. 1, Fig. 2). The adjusted HR comparing a vitamin D z-score to the mean vitamin D z-score increased as the vitamin D z-score decreased (Fig. 1). In contrast, the adjusted HR comparing a CRP z-score to the mean CRP z-score increased as the CRP z-score increased (Fig. 2). There was a significant interaction between vitamin D deficiency and CRP z-score (p < 0.001). The hazard ratio indicated that risk of diabetes was elevated (greater than 1) among those with vitamin D deficiency, regardless of CRP z-scores, but was highest among participants with lower CRP z-scores (Fig. 3).

Similar associations between vitamin D or CRP and incident diabetes were found in younger and older adults and in normal and frail or pre-frail older adults (Figures S2 and S3). The hazard ratio associated with vitamin D deficiency for incident diabetes was similar between younger and older adults regardless of CRP z-scores, but was significantly higher at low z-scores of CRP comparing frail or pre-frail older adults to older adults who were non-frail (Figure S4).

## 4 Discussion

Using data from the UK Biobank cohort, we showed inverse and positive associations of vitamin D and CRP levels with incident diabetes in ambulatory community-dwelling middle-aged and older adults. Our analyses also demonstrated significant interactions between levels of CRP and vitamin D deficiency in incident diabetes.

The lower hazard ratio (HR) associated with vitamin D deficiency for incident diabetes at higher CRP levels may indicate a role of inflammation in modifying the effect of vitamin D deficiency. The mechanism of such interaction between vitamin D deficiency and inflammation on adverse clinical outcomes may involve the shutdown of T-cell mediated inflammation by vitamin D [27]. Complement-induced vitamin D-receptor signaling switches off T helper 1 cell responses of pro-inflammatory programs.

In participants with higher levels of inflammation based on CRP, the association between vitamin D deficiency and incident diabetes was not as strong as in participants with lower levels of inflammation. This implies that without considering inflammation, correction of vitamin D deficiency alone may not yield the actual benefits of supplementation. Inflammation may have more substantial effects on diabetes risk than vitamin D deficiency (as suggested in Fig. 1, Fig. 2). This finding suggests that with higher inflammation, vitamin D deficiency may no longer have much added effect on diabetes risk. This finding is relevant when we consider vitamin D supplementation trials that showed no beneficial effects of vitamin D supplements in preventing diabetes [8], cardiovascular disease or cancer [28]. To compensate for the effects of inflammation, interventions may include increasing the dosage of vitamin D, which may improve inflammatory states, or combine vitamin D supplementation with other treatments to lessen inflammation concurrently.

Of note, a recent article reported that more than age, frailty plays a crucial role in diabetes prognosis and individualized patient care [29]. Our results support this concept by showing a stronger interaction between vitamin D deficiency and CRP on incident diabetes in pre-frail or frail older adults as compared to the non-frail. For people who become more frail and have severe vitamin D deficiency, an individualized plan of care would likely be more effective in preventing the development of diabetes. Our data support and emphasize such a precision medicine approach as a potential means of obtaining better clinical outcomes [30]. Our study also highlights the need to focus on people with vitamin D deficiency (13% in the current cohort) who may have concurrent inflammation. These people often have additional unhealthy lifestyle factors that are associated with increased diabetes risk. They may need particular attention in terms of diabetes prevention.

The UK Biobank is one of the largest cohorts globally, providing extensive clinical data linked with the UK National Health Services (NHS). This linkage enabled us to conduct a prospective cohort study using long-term follow-up data on diabetes, thereby reducing reverse causation bias. However, there are several limitations to consider. Firstly, the participants are predominantly Caucasian, healthy, and ambulatory, so caution is needed when generalizing the findings to different populations. Secondly, vitamin D and CRP levels may change over time, which may impact the effect of their interaction on diabetes risk. The potential time-dependent interaction cannot be investigated using single vitamin D and CRP measurements from baseline assessments.

Lastly, our study also has important implications from a geroscience perspective. Inflammaging, a natural biological aging process associated with immune dysregulation, varies in each individual depending on multiple health risk factors and heterogeneity in aging trajectories [31]. Although low levels of 25-OH vitamin D represent a robust predictor of many future chronic diseases, frailty, and disability, well-conducted randomized controlled clinical trials have failed to demonstrate consistent benefit [32]. Both vitamin D deficiency and inflammation become more prevalent with aging, and therefore, more older adults will likely experience both conditions simultaneously. Although vitamin D supplementation trials have failed to show consistent benefits, invoking a potential role for uncorrected or partially corrected co-existing inflammation in the benefits of vitamin D supplementation may help guide future research designs. In addition to evidence of inflammation predicting low vitamin D levels, vitamin D deficiency is associated with greater inflammation [17]. While more mechanistic studies are needed, such mutually inhibitory interactions between inflammation and vitamin D deficiency may result in enhanced or even synergistic effects on clinical outcomes, in other words, accelerate the pace of aging. A multidisciplinary approach and research from a geroscience perspective [33] may offer a more promising way of tackling this complicated issue by considering multiple risk factors simultaneously and their interaction rather than focusing on each risk factor separately, as most studies are done currently. In addition, given that different hallmarks of aging are interconnected, the scope of this study needs to be extended to see the overall picture. Geroscience-guided intervention may offer an alternative to conventional vitamin D supplementation [34]. More studies also need to be undertaken to confirm whether there are similar interactions on other adverse health outcomes in addition to the diabetes risk shown in this study. This would widen the scope of the applicability of findings from this study [35].

In conclusion, the risk of diabetes associated with vitamin D deficiency is modified by levels of concurrent inflammation. Our findings can help guide the designs of future clinical trials on vitamin D supplementation.

## CRediT authorship contribution statement

JK, GAK, and CLK designed the study, SZ and CLK processed and analyzed the data. JK, SZ, and CLK wrote the paper. GAK, LB, RF, BK, and GD reviewed the paper and provided intellectual input. All the authors reviewed and approved the final version.

## Funding information

Access to UK Biobank data was granted under application no. 92647 “Research to Inform the Field of Precision Gerontology” (PI: Richard H. Fortinsky), funded by the Claude D. Pepper Older American Independence Centers (OAIC) program: P30AG067988 (MPIs: George A. Kuchel and Richard H. Fortinsky). CLK, RHF, and GAK are partially supported by P30AG067988.

## Data availability statements

Data access is granted upon application to the UK Biobank. The R code (Liu & Kuo n.d.) for computing HPS can be obtained from the GitHub repository at https://github.com/kuo-lab-uchc/HPS.

## Declaration of competing interest

We have no conflicting interests to disclose.
