# Supplementary material for: Interactions between vitamin D deficiency and inflammation on diabetes risk: data from 336,500 UK Biobank adults
Source: J Nutr Health Aging. 2024 Dec 10;29(2):100446. doi: 10.1016/j.jnha.2024.100446 (PMC12179973; doi:10.1016/j.jnha.2024.100446)
Supplement: Supplementary file 1 [file mmc1.docx]

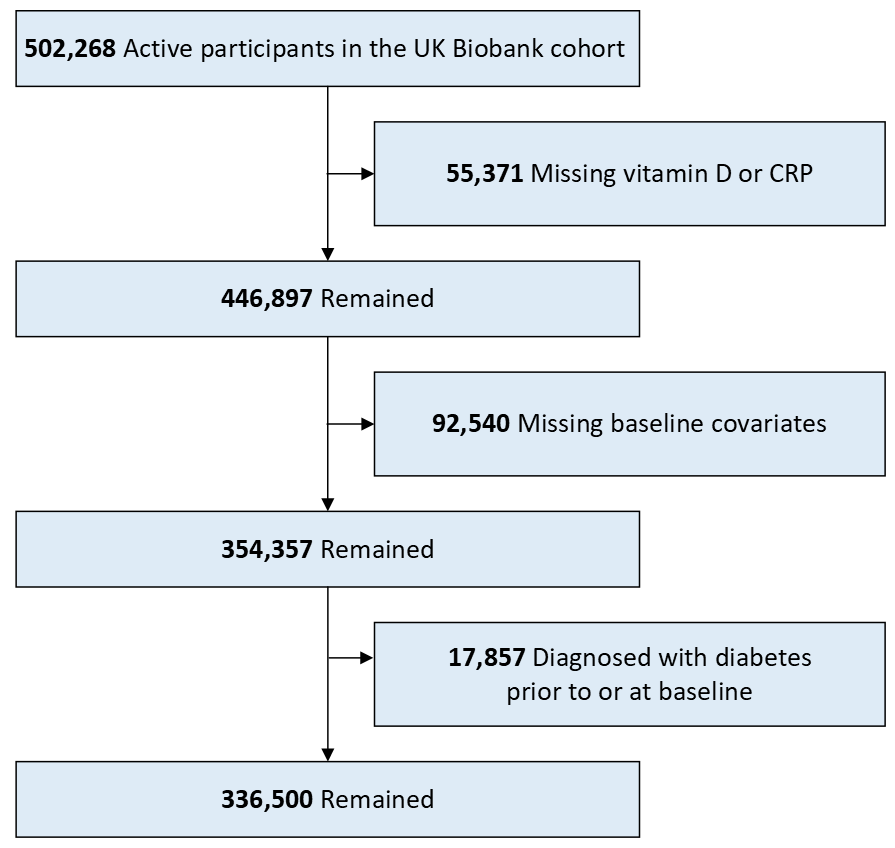


**Supplementary Figure 1 Consort flow diagram of the sample selection**

**Baseline covariates included: age, sex, ethnicity, recruitment season, education, Townsend deprivation index, smoking status, IPAQ activity group, alcohol intake frequency, BMI, vitamin D supplement, MACE status, and chronic kidney disease status.**

**
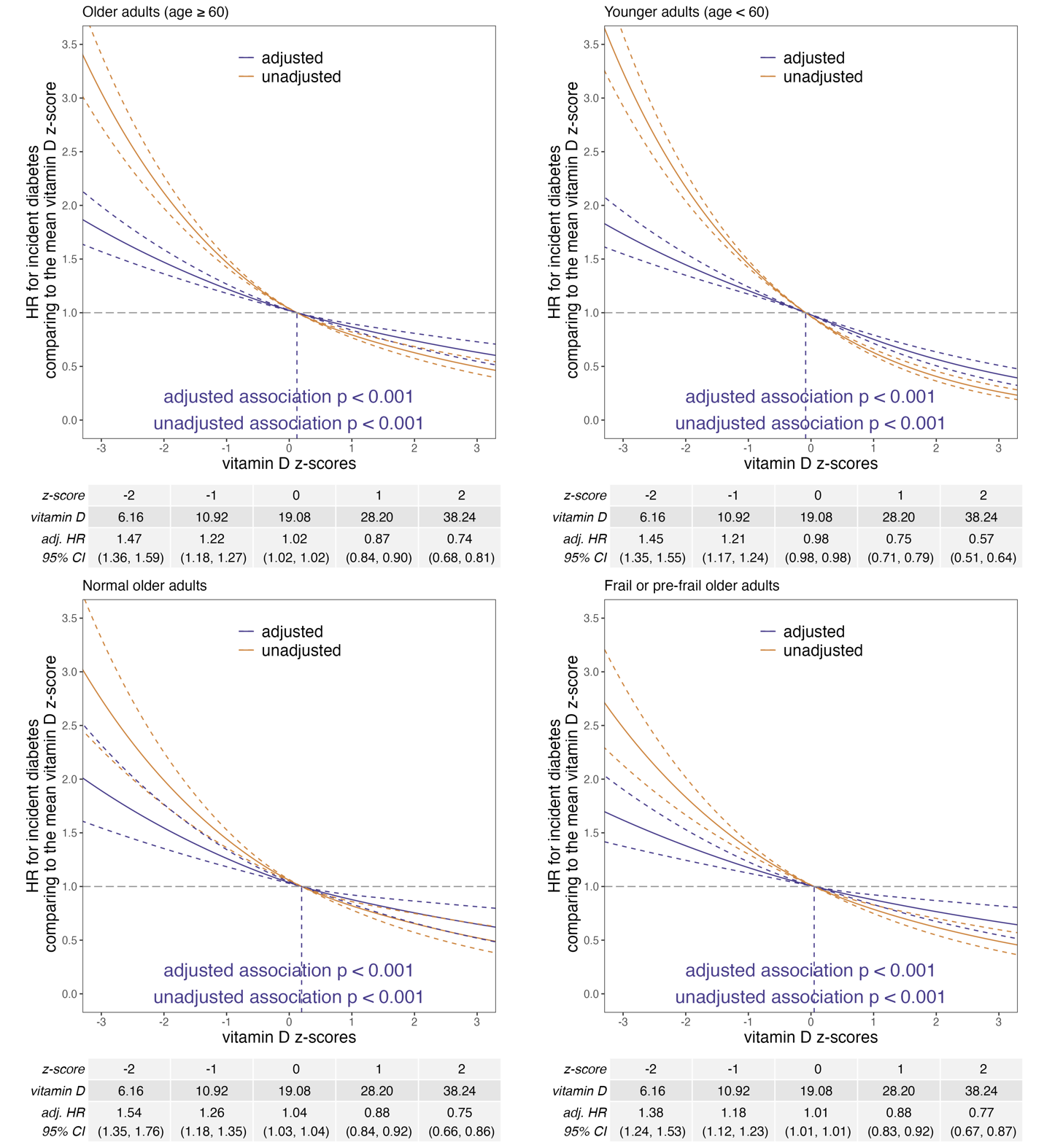
Supplementary Figure 2 Unadjusted and adjusted hazard ratios for incident diabetes comparing a vitamin D z-scores to the mean z-score in subgroups by age group and Fried frailty status. Mean vitamin D z-score: 0.12 (older adults), -0.09 (younger adults), 0.20 (non-frail older adults), 0.05 (frail or pre-frail older adults). Adjusted baseline covariates: age, sex, ethnicity, recruitment season, education, Townsend deprivation index, smoking status, IPAQ activity group, alcohol intake frequency, BMI, vitamin D supplement status, MACE status, and chronic kidney disease status.**

**
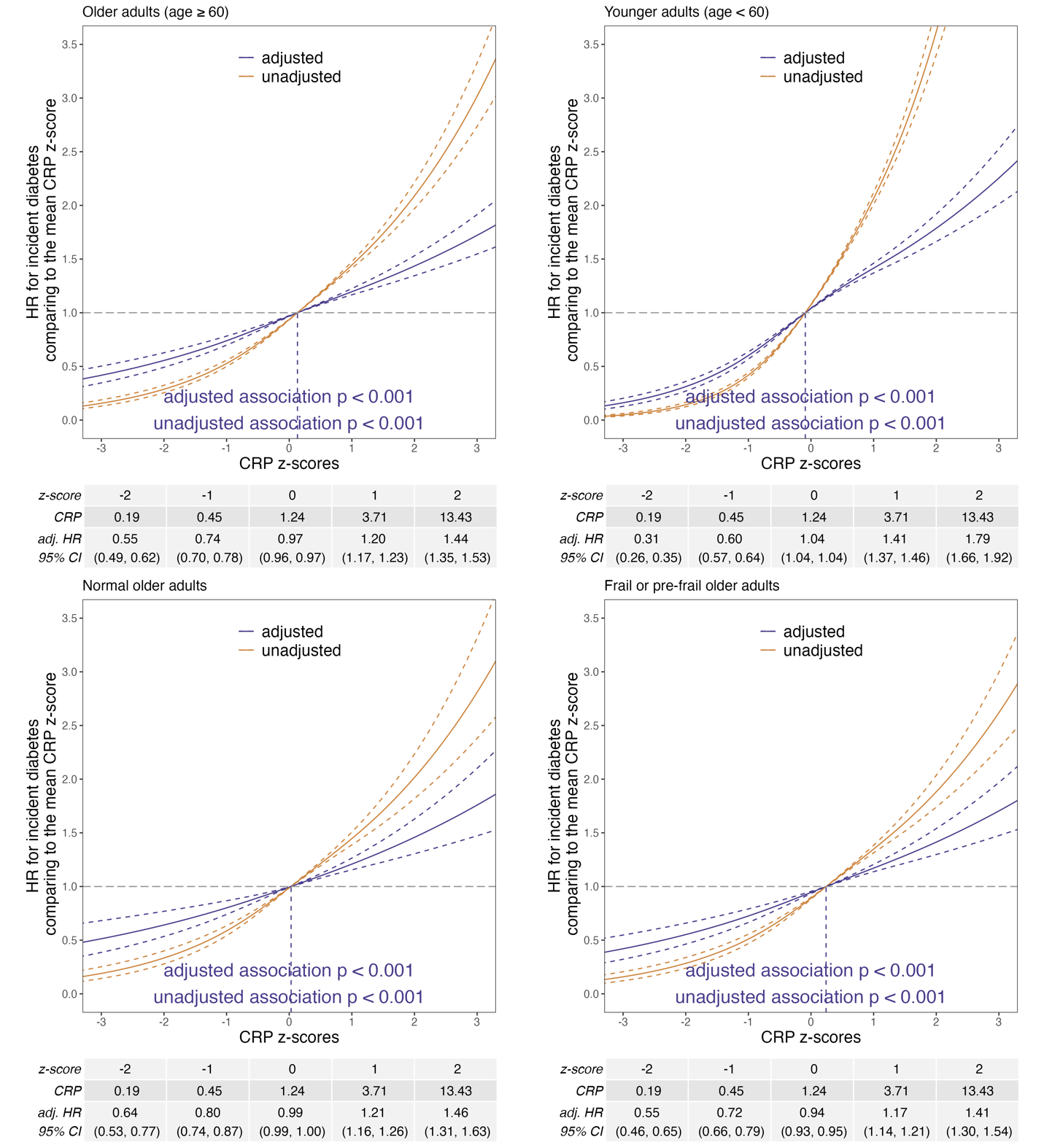
Supplementary Figure 3 Unadjusted and adjusted hazard ratios for incident diabetes comparing a CRP z-score to the mean CRP z-score in subgroups by age group and Fried frailty status. Mean CRP z-score: 0.13 (older adults), -0.09 (younger adults), 0.03 (non-frail older adults), 0.24 (frail or pre-frail older adults). Adjusted baseline covariates: age, sex, ethnicity, recruitment season, education, Townsend deprivation index, smoking status, IPAQ activity group, alcohol intake frequency, BMI, vitamin D supplement status, MACE status, and chronic kidney disease status.**

**
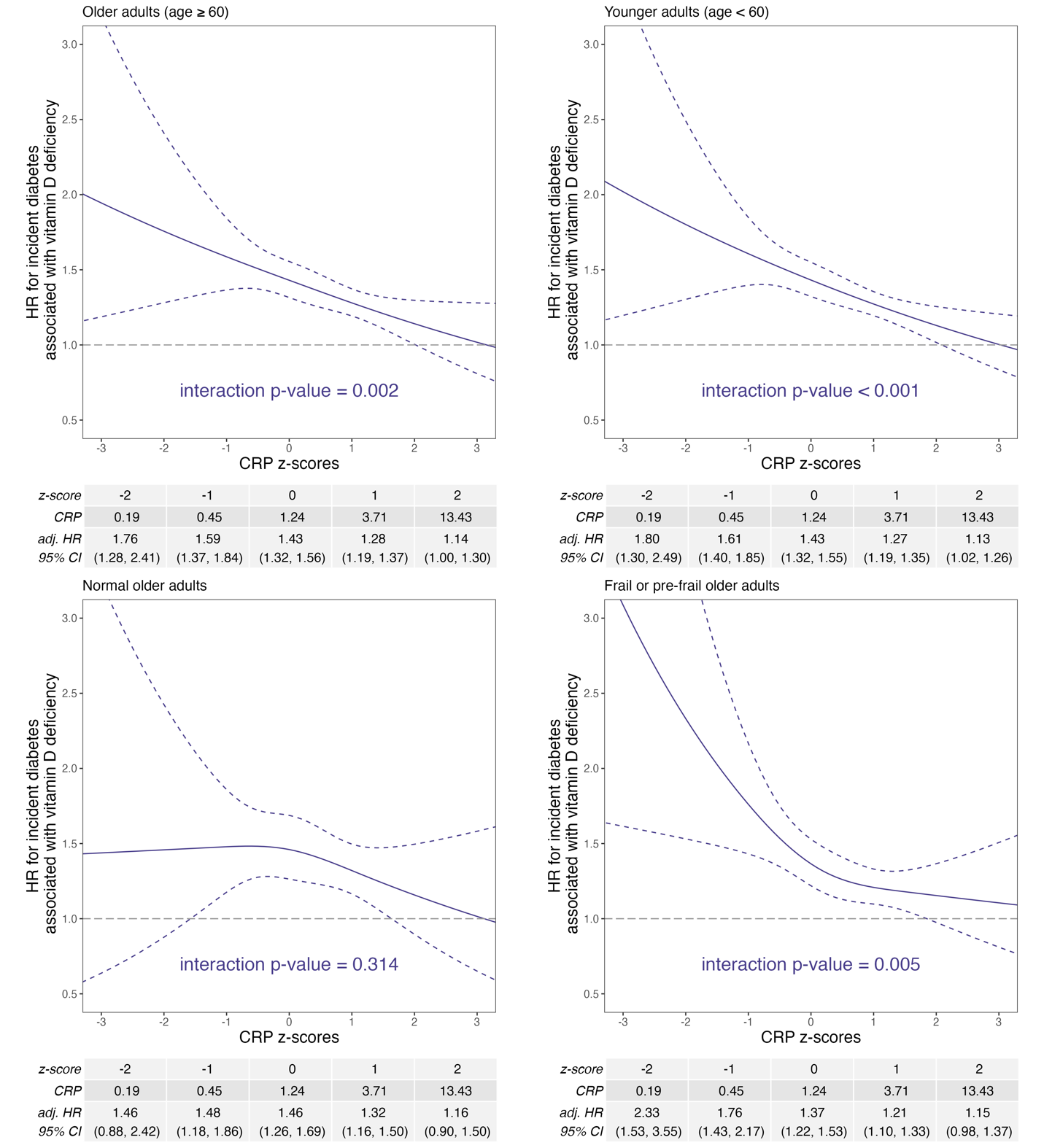
Supplement Figure 4: Association between vitamin D deficiency and incident diabetes at a given level of CRP z-score in subgroups by age group and Fried frailty status. Adjusted baseline covariates: age, sex, ethnicity, recruitment season, education, Townsend deprivation index, smoking status, IPAQ activity group, alcohol intake frequency, BMI, vitamin D supplement status, MACE status, and chronic kidney disease status.**

**Table S1 A list of UK Biobank field IDs to extract data**

| **Category** | **Description** | **Field ID** | **Instance^1^** | **ICD-10 codes (if applicable** |
| --- | --- | --- | --- | --- |
| Socio-demographics | Age at recruitment | 21022 | NA |  |
| Socio-demographics | Sex | 31 | NA |  |
| Socio-demographics | Ethnic background | 21000 | NA |  |
| Socio-demographics | Qualifications | 6138 | 0 |  |
| Socio-demographics | Month of attending assessment centre | 55 | 0 |  |
| Baseline characteristics | Townsend deprivation index at recruitment | 22189 | NA |  |
| Lifestyle and environment | IPAQ activity group | 22032 | NA |  |
| Lifestyle and environment | Alcohol intake frequency | 1558 | 0 |  |
| Lifestyle and environment | Smoking status | 20116 | 0 |  |
| Physical measures | Usual walking pace | 924 | 0 |  |
| Physical measures | Body mass index (BMI) | 21001 | 0 |  |
| Physical measures | Hand grip strength (left) | 46 | 0 |  |
| Physical measures | Hand grip strength (right) | 47 | 0 |  |
| Self-reported medical conditions | Vitamin and mineral supplements | 6155 | 0 |  |
| Self-reported medical conditions | Weight change compared with 1 year ago | 2306 | 0 |  |
| Experience of pain | Feeling tired or having little energy over the last two weeks | 120107 | NA |  |
| Blood biochemistry | Vitamin D | 30890 | 0 |  |
| Blood biochemistry | C-reactive protein | 30710 | 0 |  |
| Diabetes | Date E10 first reported (insulin-dependent diabetes mellitus) | 130706 | NA | E10 |
| Diabetes | Date E11 first reported (non-insulin-dependent diabetes mellitus) | 130708 | NA | E11 |
| Diabetes | Date E12 first reported (malnutrition-related diabetes mellitus) | 130710 | NA | E12 |
| Diabetes | Date E13 first reported (other specified diabetes mellitus) | 130712 | NA | E13 |
| Diabetes | Date E14 first reported (unspecified diabetes mellitus) | 130714 | NA | E14 |
| Major cardiovascular events (MACE) | Date I20 first reported (angina pectoris) | 131296 | NA | I20 |
| MACE | Date I21 first reported (acute myocardial infarction) | 131298 | NA | I21 |
| MACE | Date I22 first reported (subsequent myocardial infarction) | 131300 | NA | I22 |
| MACE | Date I23 first reported (certain current complications following acute myocardial infarction) | 131302 | NA | I23 |
| MACE | Date I24 first reported (other acute ischaemic heart diseases) | 131304 | NA | I24 |
| MACE | Date I25 first reported (chronic ischaemic heart disease) | 131306 | NA | I25 |
| MACE | Date I61 first reported (intracerebral haemorrhage) | 131362 | NA | I61 |
| MACE | Date I62 first reported (other nontraumatic intracranial haemorrhage) | 131364 | NA | I62 |
| MACE | Date I63 first reported (cerebral infarction) | 131366 | NA | I63 |
| MACE | Date I64 first reported (stroke, not specified as haemorrhage or infarction) | 131368 | NA | I64 |
| MACE | Date I70 first reported (atherosclerosis) | 131380 | NA | I70 |
| MACE | Date I73 first reported (other peripheral vascular diseases) | 131386 | NA | I73 |
| Chronic kidney disease | Date N18 first reported | 132032 | NA | N18 |

**^1^Instance 0: Initial assessment visit (2006-2010) at which participants were recruited and consent given; NA: not applicable**

**Table S2 Baseline participant characteristics of the included samples**

| **Variable** | **N = 336,500***^1^* |
| --- | --- |
| **Exposure** |  |
| **C-reactive Protein (mg/L)** | 2.44 (4.19); 1.24 (0.62, 2.57) |
| **Vitamin D (ng/mL)** | 20 (8); 19 (13, 25) |
| **Vitamin D deficiency (< 10 ng/mL)** |  |
| No | 294,376 (87.5%) |
| Yes | 42,124 (12.5%) |
| **Demographic Factors** |  |
| **Age at recruitment** | 56 (8); 57 (49, 63) |
| **Age Group** |  |
| <60 | 198,934 (59%) |
| ≥ 60 | 137,566 (41%) |
| **Season At Recruitment** |  |
| Spring | 96,624 (29%) |
| Summer | 89,116 (26%) |
| Fall | 82,147 (24%) |
| Winter | 68,613 (20%) |
| **Sex** |  |
| Male | 159,464 (47%) |
| Female | 177,036 (53%) |
| **Ethnicity** |  |
| White | 321,655 (96%) |
| Asian | 5,806 (1.7%) |
| Black or Black British | 4,496 (1.3%) |
| Other | 4,543 (1.4%) |
| **Socioeconomic Factors** |  |
| **Townsend Deprivation Index** | -1.46 (3.00); -2.27 (-3.70, 0.25) |
| **Education***^2^* |  |
| None of the above | 47,449 (14%) |
| Other professional qualifications eg: nursing, teaching | 16,944 (5%) |
| A-levels/NVQ/HND/HNC | 61,839 (18%) |
| GCSEs/O-levels | 71,963 (21%) |
| CSEs or equivalent | 17,617 (5%) |
| College or University degree | 120,688 (36%) |
| **Lifestyle Factors** |  |
| **Vitamin D Supplement Status** |  |
| No | 330,384 (98%) |
| Yes | 6,116 (1.8%) |
| **Smoking Status** |  |
| Never | 186,354 (55%) |
| Previous | 116,328 (35%) |
| Current | 33,818 (10%) |
| **Alcohol Frequency** |  |
| Never | 23,063 (6.9%) |
| Special occasions only | 34,497 (10%) |
| One to three times a month | 36,642 (11%) |
| Once or twice a week | 87,567 (26%) |
| Three or four times a week | 82,313 (24%) |
| Daily or almost daily | 72,418 (22%) |
| **IPAQ Activity Group** |  |
| Low | 61,290 (18%) |
| Moderate | 137,400 (41%) |
| High | 137,810 (41%) |
| **Physical Measures** |  |
| **BMI** | 27.1 (4.5); 26.5 (24.0, 29.5) |
| **Fried Frailty (age ≥ 60 only)** |  |
| Frail or Pre-Frail | 55,718 (43%) |
| Normal | 73,245 (57%) |
| **Disease Status** |  |
| **MACE Status** |  |
| No | 314,431 (93%) |
| Yes | 22,069 (6.6%) |
| **Chronic Kidney Status** |  |
| No | 333,275 (99%) |
| Yes | 3,225 (1%) |
| *^1^*Mean (SD); Median (IQR) or Frequency (%) | |
| *^2^*Education: CSE - Certificate of Secondary Education; GCSE - General Certificate of Secondary Education; O-levels - Ordinary Levels; A-levels - Advanced Levels; NVQ - National Vocational Qualification; HND - Higher National Diploma; HNC - Higher National Certificate | |
